# Small, Dense Low-Density Lipoprotein-Cholesterol and Atherosclerosis: Relationship and Therapeutic Strategies

**DOI:** 10.3389/fcvm.2021.804214

**Published:** 2022-02-10

**Authors:** Xiao Jin, Shengjie Yang, Jing Lu, Min Wu

**Affiliations:** ^1^General Department of Guang'anmen Hospital, China Academy of Chinese Medical Sciences, Beijing, China; ^2^Beijing University of Chinese Medicine, Beijing, China

**Keywords:** small dense low-density lipoprotein-cholesterol, atherosclerosis, lipid metabolism, inflammation, endothelial injury, review

## Abstract

Low-density lipoprotein cholesterol (LDL-C) plays an important role in the formation, incidence, and development of atherosclerosis (AS). Low-density lipoproteins can be divided into two categories: large and light LDL-C and small, dense low-density lipoprotein cholesterol (sdLDL-C). In recent years, an increasing number of studies have shown that sdLDL-C has a strong ability to cause AS because of its unique characteristics, such as having small-sized particles and low density. Therefore, this has become the focus of further research. However, the specific mechanisms regarding the involvement of sdLDL-C in AS have not been fully explained. This paper reviews the possible mechanisms of sdLDL-C in AS by reviewing relevant literature in recent years. It was found that sdLDL-C can increase the atherogenic effect by regulating the activity of gene networks, monocytes, and enzymes. This article also reviews the research progress on the effects of sdLDL-C on endothelial function, lipid metabolism, and inflammation; it also discusses its intervention effect. Diet, exercise, and other non-drug interventions can improve sdLDL-C levels. Further, drug interventions such as statins, fibrates, ezetimibe, and niacin have also been found to improve sdLDL-C levels.

## Introduction

Atherosclerosis (AS) is the formation of fibrofatty lesions within the arterial wall, and it causes widespread morbidity and mortality worldwide together with heart muscle infarction, stroke, and disabling peripheral artery illness (1). AS could be a major condition that seriously harms human health and is understood as the major cause of mortality not only in developed countries, but globally (2). At present, inflammatory reactions (3), lipid metabolism disorders (4), and oxidative stressare the most important and widely recognized pathogenic causes of AS (5). In the field of lipid metabolism, a number of irrefutable pieces of evidence have proven the pathogenic role of low-density lipoprotein cholesterol (LDL-C) in AS, so we have extremely effective tools to reduce LDL-C levels, thus reducing the occurrence of cardiovascular events (2). Some studies have shown the correlation between different low-density lipoprotein (LDL) subgroups and the occurrence of AS, in which small, dense low-density lipoprotein cholesterol (sdLDL-C) is closely related to and has a stronger effect on AS (6). This study aimed to explore the relationship between sdLDL-C levels and AS.

## Detection Method and Sources of SdLDL-C

**Figure 1:**
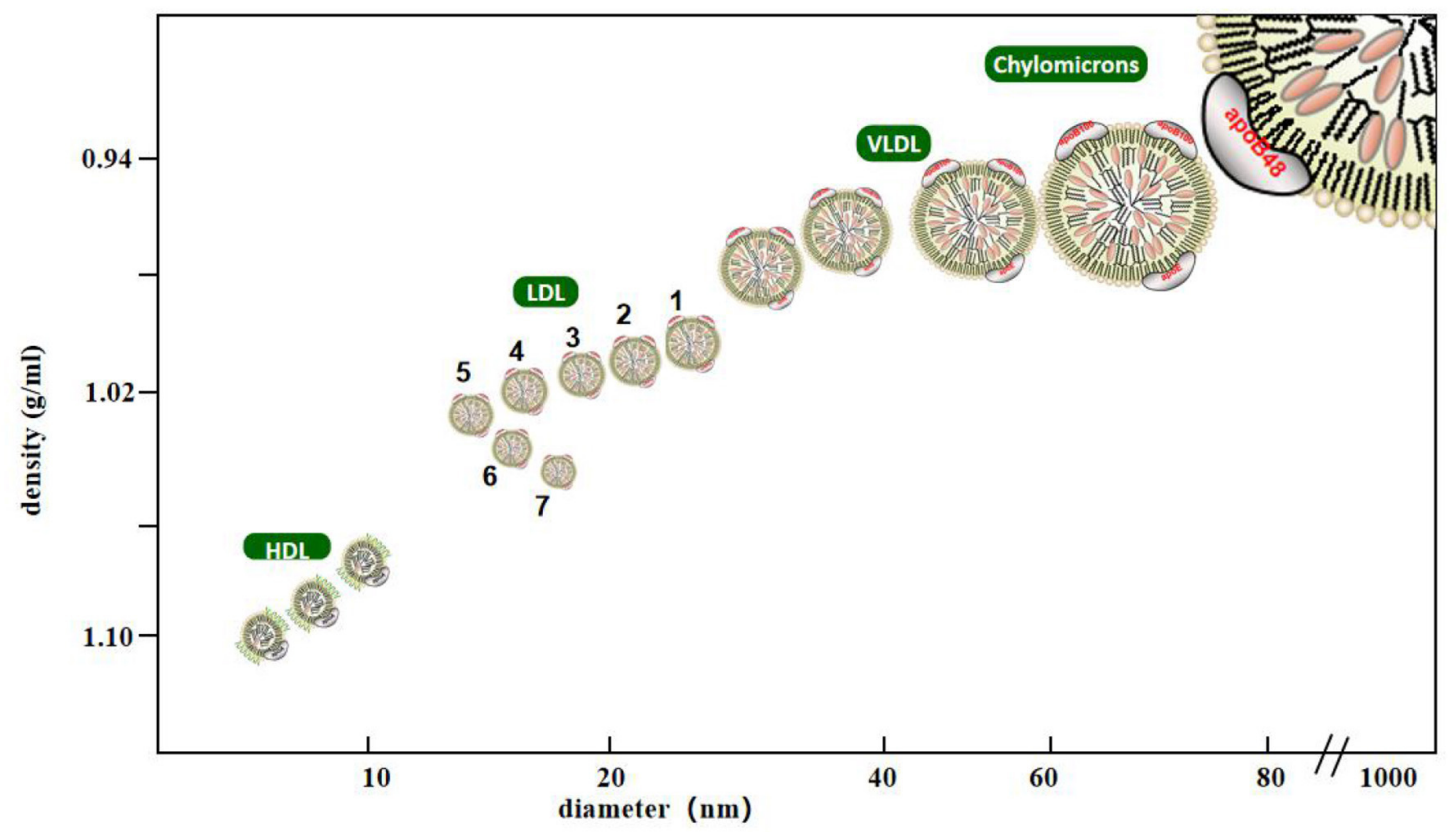
Classification of lipoprotein cholesterol. Lipoprotein with density <0.95 g/ml, diameter of 80–500 nm is chylomicrons, density of 0.95–1.006 g/ml, diameter of 25–80 nm is very low density lipoprotein, density of 1.063–1.21 g/ml, diameter of 8–15 nm is high density lipoprotein, density of 1.006–1.063 g/ml, diameter of 18–28 nm is low density lipoprotein, Among them, low-density lipoprotein is divided into seven subtypes. The third to seventh subtypes are small and dense low-density lipoprotein with density >1,004 g/ml and diameter <25.5 nm.

LDL is a lipoprotein with a density between 1.006 and 1.063 g/mL. It is composed of many heterogeneous particles. They can be separated using various experimental methods. Krauss et al. used ultracentrifugation to classify LDL into four types according to density: large and light LDL- I (1.025–1.034 g/mL), intermediate density LDL- II (1.035–1.044 g/mL), low-density LDL- III (1.045–1.060 g/mL), and very low-density LDL-IV (7). Another method widely used to identify low-density lipoproteins is gradient gel electrophoresis (GGE), which separates low-density lipoprotein particles by electrophoretic mobility. In studies using GGE, LDL particles have been separated into four major subfractions, LDL I (large LDL, peak diameter 26.0–28.5 nm), LDL II (intermediate LDL, 25.5–26.4 nm), LDL III A and B (small LDL, 24.2–25.5 nm), and LDL IV A and B (very small LDL, 22.0–24.1 nm) (8). The Lipoprint LDL subcomponent rapid analysis system (Hitachi 7180) is the only diagnostic equipment certified by the United States Food and Drug Administration for the separation and detection of LDL subcomponents. Based on the charge and particle size of LDL-C, they can be divided into seven subcomponents within a short time via polyacrylamide gel electrophoresis, in which components 3–7 were defined as sdLDL-C (Figure 1) (9). In addition, foreign enzyme-linked immunosorbent assay (ELISA, Millipore, St. Louis, MO) kits can quickly detect the concentrations of sdLDL-C but do not rule out the possibility of cross-reactions, so it remains to be verified whether they can be used in clinical settings (10). The homogeneous method (11) can be used to measure sdLDL-C levels by removing lipoproteins other than sdLDL-C using a surfactant and sphingomyelinase, and it is a better technique compared to the traditional detection method of LDL subfraction. Its correlation, accuracy, and stability are high, which provided a cornerstone for the popularization and application of clinical sdLDL-C detection. Other analytical methods to detect sdLDL-C include gel filtration column chromatography, high performance liquid chromatography (HPLC), ion mobility analysis, and dynamic light scattering. In clinical practice, it is important to accurately analyze LDL subclasses thorough analytical methods.

For the detection of sdLDL-C, Mauree et al. developed a new equation to calculate the content of sdLDL-C {the formula of sd-LDL-C is as follows: ElbLDL-C = 1.43 × LDL-C −[0.14 × (ln (TG) × LDL-C)] − 8.99; EsdLDL-C = LDL-C−ElbLDL-C}. Equations for sdLDL-C was generated with least-squares regression analysis using the direct Denka sdLDL-C assay as reference (*n* = 20,171). This equation can be widely used for all patients with standard lipid groups without incurring additional laboratory testing costs. The limitation of the study is that the study population is not universal. The equation developed is based on fasting subjects. Therefore, whether this equation can be used in experiments or clinical applications remains to be verified (12).

About the detection method of sdLDL-C (8), ultracentrifugation involves multiple steps and requires specialized equipment and expertise to separate LDL subsets, which is more error-prone, time-consuming, laborious and costly. The resolution of gradient gel electrophoresis is very high, but it requires a lot of manpower, material and financial resources, and the sample flux is low. The polyacrylamide gel tube electrophoresis method is simple to operate and shows a satisfactory coefficient of variation between samples, but the disadvantage is that the equipment is expensive. The homogeneous method can be fully automated and can be used in the high-throughput integration platform, which is helpful for the large-scale testing of sdLDL-C.

At present, it is generally believed that there are two ways to produce sdLDL-C (7, 13). First, when the triglyceride (TG) content in the liver is high, the liver directly secretes VLDL1 (large particles and high TG content) and VLDL2 (low TG content), when the level of TG synthesized by hepatocytes decreases, the liver secretes VLDL1 (small particles and high TG content) and intermediate density lipoprotein2 (low TG content). TG-deficient lipoproteins are the precursors of larger LDL (LDL I and LDL II), while TG-rich lipoproteins are converted into sdLDL (LDL III and LDL IV) after being defatted by lipoprteinlipase (LPL) and hepatic lipase (HL). Second, there is a very active and dynamic lipid exchange between various lipoproteins in the plasma, which is mainly catalyzed by cholesterol lipid transport proteins. The total cholesterol (TC) of LDL is transferred to VLDL and the TG of VLDL is transferred to LDL, but the total amount and synthesis of LDL remains unchanged. When the TG levels in LDL increase to a certain extent, LDL will be hydrolyzed by liver lipase to remove TG, the LDL particles become smaller, then the TC content decreases, thus promoting the formation of sdLDL-C. When the plasma concentration of TG exceeds 1.5 mmol/L, lipid exchange is accelerated; higher TG levels accelerate the lipid interaction between VLDL and LDL, producing more sdLDL-C. It has been reported in the literature that the change in gene locus is also significantly related to sdLDL-C levels. In 2009, Musunuru et al. (14) proved that four genes, *CEPT, LIPC, APOA1/A5*, and *LPL* are related to the particle size and distribution of LDL or HDL. Hoogeveen (15) showed that 127 single nucleotide polymorphisms (SNPs) are considerably related to sdLDL-C. These SNPs are distributed in eight different sites on chromosomes 1, 2, 7, 8, 11, and 19, and are distributed in 14 different genes. The genetic variation of these genes is related to lipid metabolism and inflammatory pathways. This study also found that the genetic variation of the new locus *PCSK7* was also associated with sdLDL-C levels.

## Relationship Between SdLDL-C and AS

AS mainly affects the intima of the large- and medium-sized arteries, characterized by lipid deposition, focal fibrosis, and the formation of atherosclerotic plaques, resulting in thickening, hardening, and lumen stenosis of the vessel wall, ultimately leading to ischemic changes in the corresponding organs. In recent years, some studies have confirmed that the ability of LDL-C to induce AS varies with different densities, and there is a stronger relationship between sdLDL-C and the stability of AS plaques (16, 17). Ikezaki (18) followed 2,030 men and women [median age 59 years old, no cardiovascular disease (CVD) and not taking cholesterol-lowering drugs] for five years and performed univariate, multivariate regression and least squares analysis to examine the relationship between direct sdLDL-C and other lipoproteins with the progression of carotid intimal medial thickness (cIMT). The plasma levels of direct sdLDL-C and other lipoproteins were measured using a homogeneous detection kit obtained from Denka-Seiken. The results showed that compared with LDL-C, sdLDL-C had a stronger correlation with the progress of cIMT. However, the scale of this line-up study is small and all subjects are Japanese. A larger population of different races is needed to verify this study.

Duran et al. conducted a prospective case cohort study to study the relationship between sdLDL-C and cardiovascular events. The sdLDL-C concentration in this study was directly measured using a two-stage automatic homogenization test also developed by Denka Seiken Co (Niigata, Japan). A total of 27,552 participants provided sufficient blood samples when they entered the group after taking into account the missing key exposure data, the final sample included 480 women with total CVD and 496 women whose age and smoking frequency matched. The study found that the concentration of sdLDL-C in women with myocardial infarction (MI) was much higher than that in the control group, suggested that there was a significant correlation between plasma sdLDL-C concentration and MI (17).

Balling et al. measured sdLDL cholesterol using Denka Seiken's assay in 38,322 individuals participating in the Copenhagen General Population Study from 2013 to 2017. The death and immigration information comes from the Danish civil registration system. Individuals are followed up from baseline to December 2018 for MI and atherosclerotic cardiovascular disease (ASCVD). events, death, immigration, or the end of follow-up, whichever occurs first. Covariates were measured at baseline, including smoking, lipid-lowering therapy, blood pressure, body mass index, diabetes, blood sample analysis, total cholesterol, high-density lipoprotein cholesterol, triglycerides and apolipoprotein B. The Cox regression restricted cubic spline model with multivariate adjustment was also used to examine the association between sdLDL cholesterol and the risk of MI. The median follow-up time was 3.1 years. In the multivariate adjusted spline curve, as the concentration of sdLDL cholesterol increased, the risk of myocardial infarction was observed to increase (6). A large amount of experimental data (9, 15, 19–25) showed that sdLDL-C is closely related to the occurrence of cardiovascular events, so it is necessary to monitor clinically and reduce the concentration of sdLDL-C to reduce the occurrence of cardiovascular events (Table 1).

**Table 1:** The relationship between sdLDL-C and AS.

**References**	**Research type**	**Detection method of sdLDL-C**	**Number of participants**	**Age**	**Follow-up time/years**	**Conclusions**
Tsai et al. (9)	Retrospective analysis	Homogenous assay	4,387 atherosclerotic participants	Not mentioned	8.5	sdLDL-C was associated with CVD
Hoogeveen et al. (15)	Retrospective analysis	Homogenous assay	9,882 atherosclerotic participants	45–64	11	sdLDL-C was associated with CVD
Higashioka et al. (19)	Prospective study	Homogeneous assay	3,080 without prior CVD	>40	8.3	SdLDL-C was associated with CVD
Zhou et al. (20)	Single-centre retrospective observational study	Lipoprint LDL system	368 AIS and 165 non-AIS patients	>40	None	SdLDL-C was risk factors for increased IMT
Siddiqu et al. (22)	Ancillary study	Qualitative assay kits	130 liver transplant recipients	>47	4	sdLDL-C independently predicted CVD
Goel et al. (23)	Observational, single centre, cross sectional case control study	Enzymatic analysis	150 CAD patients and 40 healthy adults	Not mentioned	None	CAD have higher sdLDL levels compared to individuals without CAD
Williams et al. (24)	Double-blind randomized controlled clinical trial	Gradient gel electrophoresis	160 patients selected for clinical coronary disease	Men <70, Women <65	2	SdLDL-C was related to changes in coronary artery stenosis and cardiovascular events in patients with CAD and low HDL-C
Arai et al. (25)	Prospective study	Homogenous assay	2030 without cardiovascular disease	Not mentioned	11.7	sd-LDL-C was significantly associated with CVD

*SdLDL-C, small, dense low-density lipoprotein cholesterol; LDL-C, low-density lipoprotein cholesterol; HDL, high-density lipoprotein; CAD, coronary artery disease; CVD, cerebrovascular disease; AIS, acute ischemic stroke*.

## Mechanism of AS Induced by SdLDL-C

The term “induration of the arteries” refers to a condition where lipids and alternative substances deposit in and on the artery walls (referred to as “plaques”) that limit traditional blood flow (26). AS and the pathology of related ischemic organs are the primary causes of mortality worldwide (27). Currently, there are many theories regarding the mechanisms of AS. It is generally believed that AS is a chronic inflammatory process involving multiple cell types and cytokines (3, 28). AS begins with the deposition of LDL-C, endothelial dysfunction, the accumulation of foam cells under the endothelium, and the formation of fat streaks through the activation of pro-inflammatory f actors (1).

### sdLDL-C Promotes AS by Regulating Lipid Metabolism

The metabolism of cholesterol esters (CEs) is regulated by the macrophage gene network. Studies have shown that genes such as *ATF3* (activating transcription factor 3) and *EGR2* affect AS by regulating lipid metabolism (29–32). *ATF3* is a member of the mammalian activation transcription factor/cAMP response element binding (CREB) family (33, 34). SdLDL-C can reduce the ability of ATF3 to induce type B scavenger receptor (SR-BI) by down-regulating the expression of ATF3, and promote hepatic cholesterol 12α hydroxylase (CYP8B1) by interacting with p53 and hepatocyte nuclear factor 4α, thus reducing the uptake of high-density lipoprotein, promoting visceral fat and cholesterol absorption, and inhibiting the reverse cholesterol transport of phagocytes (32). *EGR2* is another gene related to cholesterol metabolism, which is involved in the synthesis of free cholesterol (FC) and lipid droplets (LD) (35).

There were some differences in the content of components in different subgroups of LDL-C. SdLD-C showed a significant decrease in free cholesterol (FC), cholesterol ester (CE) and phospholipid (PL) than large and light LDL-C. The study of biofilm and lipid bilayer shows that the incorporation of cholesterol affects the permeability of metabolites. With the increase of FC content, the accessibility of oxidants to lipid core decreased, which may be a reasonable explanation for the protective effect of LDL particles on oxidation susceptibility. The increase of FC content in LDL particles may directly regulate the susceptibility to oxidative stress and help to prevent LDL particles from undergoing subsequent oxidative modification (36). Studies have shown that the introduction of sdLDL-C into traditional M2 macrophages can inhibit the expression of EGR2, resulting in the increase of CE production and FC efflux (29), therefore, the sensitivity of sdLDL-C to lipid peroxidation increased with the decrease of FC content in each particle. Ohmura evaluated the sensitivity to lipid peroxidation modification of low density lipoprotein by the formation of conjugated diene induced by copper, which also confirmed that sdLDL-C was more sensitive to lipid peroxidation modification (36). On the other hand, due to the efflux of FC, when macrophages accumulate a large amount of FC, it is a powerful apoptosis inducer, which will lead to the release of intracellular contents and thrombosis (37). In addition, studies have shown that the plasma retention time of apolipoproteinB-100 (apoB-100) on the surface of sdLDL-C is significantly longer than that of apoB-100 on the surface of IbLDL, which increased the possibility of oxidation (38). LDL is cleared after binding to LDL-C receptors, but apoB-100 on the surface of sdLDL-C molecules and LDL-C receptors has a low affinity, which makes it difficult for the receptors to recognize sdLDL-C, and is more likely to be absorbed by phagocytes, which develop into foam cells and promote the occurrence and development of AS (39).

**Figure 2:**
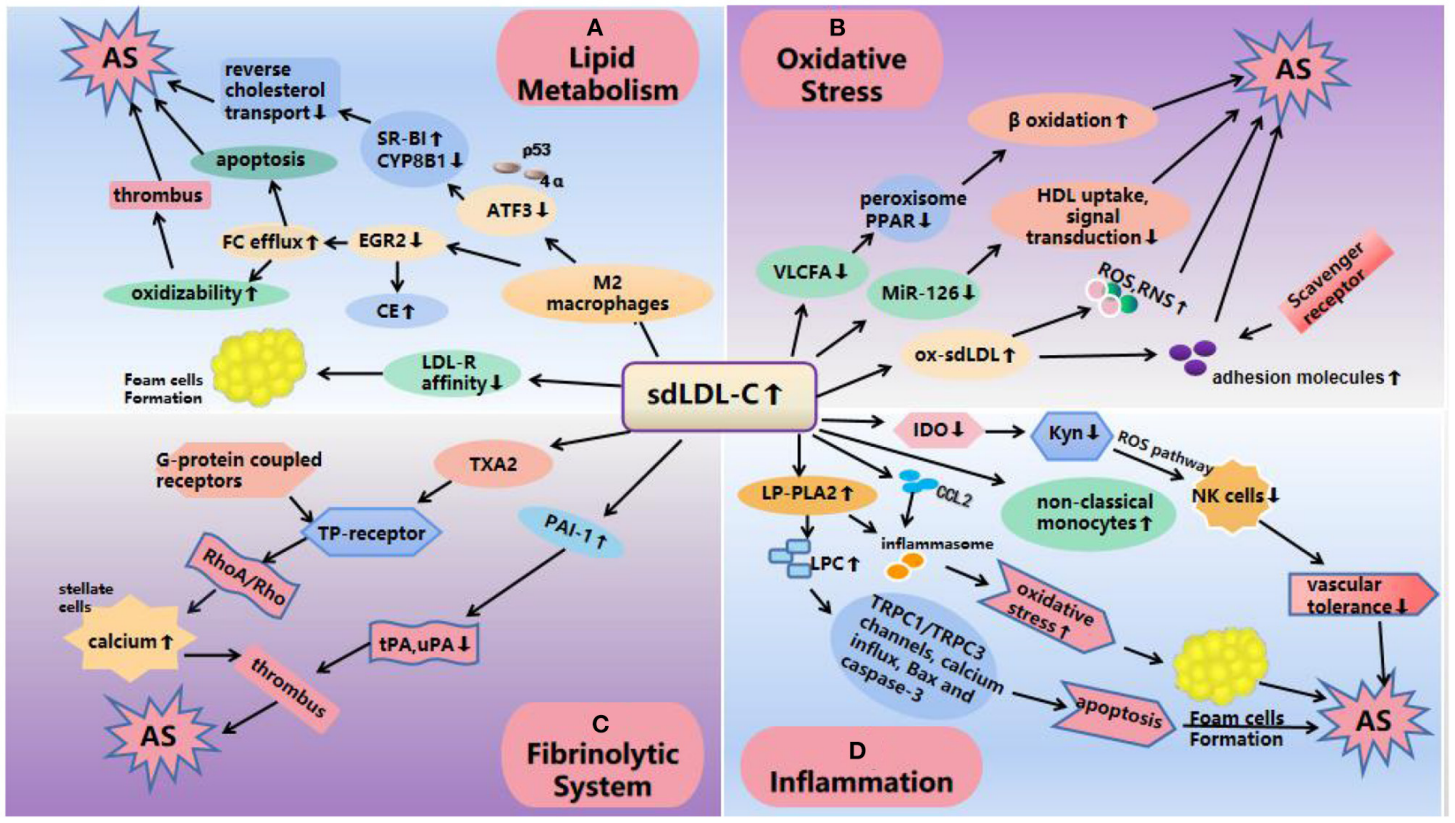
Mechanisms of atherosclerosis induced by sdLDL-C. **(A)** Lipid metabolism: SdLDL-C reduces the expression of ATF3 and EGR2, ATF3 decreases the ability of SR-BI by interacting with p53 and 4α and promotes CYP8B1 to inhibit cholesterol reverse transport; EGR2 leads to an increase in CE production and FC outflow, thus increasing the oxidation sensitivity of sdLDL-C. On the other hand, the low affinity of apoB-100 on the surface of LDL-C receptors and sdLDL-C makes it difficult for the receptors to recognize sdLDL-C and is more easily absorbed by phagocytes to form foam cells and promote the occurrence and development of AS. **(B)** Oxidative stress: The increase of sdLDL-C level reduces the production of VLCFA and miR-126, which affects lipid metabolism and fatty acid β oxidation; miR-126 affects HDL uptake, and enhances signal transduction resulting in AS. In addition, ox-sdLDL can also increase the expression of adhesion molecules and induce excessive production of ROS and RNS, resulting in the enhancement of oxidative stress to cause AS. **(C)** Fibrinolytic system: SdLDL-C increases the levels of PAI-1 and TXA2. PAI-1 inhibits the function of u-PA and t-PA, which easily leads to thrombosis. TXA2 activates TP receptor and activates RhoA/Rho21 kinase pathway through its G protein coupled receptor, and increases calcium levels in hepatic stellate cells, resulting in vasoconstriction, platelet aggregation, thrombosis and AS. **(D)** Inflammation: SdLDL-C levels reduces IDO, causing a decrease in vascular tolerance by affecting the Kyn pathway; LP-PLA2 increased that activated TRPC1/TRPC3 channels, calcium influx, Bax and caspase-3 pathways to cause apoptosis; increased expression of inflammatory cytokines and the formation of foam cell, suggesting an inflammatory response. SdLDL-C, small, dense low-density lipoprotein cholesterol; AS, atherosclerosis; CE, cholesterol ester; FC, free cholesterol; VLCFA, very-long-chain fatty acid; ATF3, activating transcription factor 3; LDs, lipid droplets; IDO, indoleamine 2,3-dioxygenase; LP-PLA2, lipoprotein-associated phospholipase A2; LPCs, lysophosphatidylcholine; ROS, reactive oxygen species; RNS, reactive nitrogen species; PAI-1, plasminogen activator inhibitor 1; TXA2, thromboxane A2; t-PA, tissue type plasminogen activator; u-PA, urokinase type plasminogen activator.

Current research shows that the increase of sdLDL-C level will reduce the generation of very-long-chain fatty acid (VLCFA), the ability to regulate peroxisome function and interact with peroxisome proliferator activated receptor (PPAR) was weakened (40), thus affecting lipid metabolism, fatty acid β oxidation, plasminogen (PL) biosynthesis and so on (41). This may also be one of the reasons why sdLDL-C has a stronger ability to cause AS. In addition, quantitative reverse transcriptase polymerase chain reaction (qRT-PCR) was used to detect serum miR-126 and 122 levels in 78 patients with CAD and 60 patients without CAD in one study. There, it was suggested that miR-126 may play a role in the cholesterol metabolism of sdLDL-C. However, the mechanism by which the decrease in circulating miR-126 levels in patients with CAD is proportional to the increase in sdLDL-C is not fully understood (42). In 2012, miRNAs were reported to be important regulators of HDL metabolism and reverse cholesterol transport, including direct targeting of cellular cholesterol efflux, HDL biogenesis, liver high-density lipoprotein uptake, and the synthesis of bile acid and secretion-related genes (43). It has also been reported that miR-126 attenuates oxidized LDL (ox-LDL)-induced endothelial cell injury by inhibiting signal transduction to delay AS (44, 45). Therefore, a larger sample cohort is needed to further explain the role of miR-126 in sdLDL-C cholesterol metabolism and to understand the development of the disease (42) (Figure 2).

### sdLDL-C Promotes AS by Inducing Inflammation

Monocytes play an important role in the early formation and maturation of plaques. They are drawn to the arteries by chemokines, such as CCL2, which are secreted by activated epithelial tissue cells (46–49) and take up lipids among the subendothelial tissue to differentiate into foam cells (50). In addition, they can also engulf precipitated cholesterol crystals (51) and oxidized lipid species (52–54) that activate the inflammasome, resulting to cell death in a highly inflammatory form called prolapse, as well as the induction of innate immune responses (51). Human monocytes are mainly divided into three types: classical (CD14^+^CD16^−^), non-classical (CD14^−^CD16^+^), and intermediate (CD14^+^CD16^+^) (55). Supported by proof from murine studies (56, 57), as well as current human observations (58), classical monocytes are believed to have the ability to differentiate into monocyte-derived macrophages and monocyte-derived dendritic cells (59) and play an indispensable role in the formation and regression of tissue inflammation. Related studies have shown that the production of sdLDL-C is related to an increase the number of non-classical monocytes and a decrease in the number of classic monocytes (60). The specific mechanism has not been detailed, but we speculate that the effect of sdLDL-C on AS may be related to the inflammatory response of monocytes. In 2017, monocytes from healthy people (stenosis degree <5%) and patients (stenosis degree >70%; single-vessel disease, two-vessel disease, three-vessel disease) were separated using a Rosette Sepkit, and macrophage colony stimulating factor (M-CSF) was used to induce them to differentiate into M2 macrophages. qRT-PCR and ELISA were used to detect MRC1 gene expression and histamine levels, respectively. After sdLDL-C treatment, the expression level of MRC1 in normal human M2 macrophages was significantly increased (*P* = 0.05), while the expression level of MRC1 gene was decreased in patients with single-vessel disease (*P* = 0.05), two-vessel disease (*P* = 0.01), and three-vessel disease (*P* = 0.9). The histamine levels secreted by M2 macrophages (after treatment for 7 day) in the case group were higher than that in the healthy control group (>3-fold, *P* = 0.02). The results illustrated that sdLDL-C granules decreased the expression of MRC1 in differentiated M2 macrophages from patients with CHD. In addition, they have a strong ability to secrete histamine (61).

Hassanpour et al. detected the effect of sdLDL-C on the changes in IDO in differentiated macrophages using RT-qPCR, colorimetric, and ELISA methods. Their results show that sdLDL-C reduces the expression and activity of IDO in macrophages (62), IDO is the first and rate-limiting enzyme in the tryptophan (Trp)-degraded kynurenine (Kyn) pathway, and its downstream metabolite is collectively called kynurenines (63). The expression of IDO was inhibited, the metabolic pathway of Trp was blocked, the formation of Kyn was decreased, the ability of cell death mediated by kyn through reactive oxygen species (ROS) pathway in natural killer (NK) cells decreased (64), vascular tolerance decreased, and promoted the occurrence and development of inflammation and AS (65, 66). In addition, ATF3 (67, 68) and EGR2 (69, 70) play crucial roles in signal transduction in the process of anti-apoptosis, anti-migration, and anti-inflammation. sdLDL-C also promotes inflammation and accelerates AS by inhibiting the expression of AFT3 and EGR2 (32).

Lipoprotein-associated phospholipase A2 (Lp-PLA2) hydrolyzes phospholipids and releases pro-inflammatory products; therefore, it is considered to be a new biomarker of vascular risk (71–74). Among the phenotypes of LDL, Lp-PLA2 preferentially binds to small, dense LDL particles (72) to produce lysophosphatidylcholine (LPC). LPC can promote the expression of inflammatory factors (75), damage arterial relaxation, increase oxidative stress, induce endothelial activation and atherosclerosis (76). SdLDL-C granules contain more Lp-PLA2. Studies have confirmed that LPC can induce apoptosis of human coronary artery smooth muscle cells by activating TRPC1/TRPC3 channels, calcium influx, Bax and caspase-3, and lead to atherosclerosis and coronary artery disease (77), which may also be a mechanism of AS induced by sdLDL-C. In addition, higher concentrations of LPC can destroy the integrity of mitochondria and enhance the release of cytochrome C in hepatocytes (78). We speculate that sdLDL-C may lead to AS through mitochondrial damage, which may be a potential mechanism (Figure 2).

### sdLDL-C Promotes AS by Enhancing Endothelial Injury

Vascular epithelial tissue cell pathology plays an important role in the initiation and development of AS (79, 80). Endothelial cell injury increases intimal permeability and leukocyte adhesion, promoting thrombus formation and rapid malady progression (81). Both ox-LDL and cholesterol cause functional damage to the arterial intima, change the surface characteristics of endothelial cells and leukocytes (monocytes and lymphocytes), and increase the expression of adhesion molecules. The number of monocytes adhering to endothelial cells increases and gets transferred from endothelial cells to subintimal macrophages, which are then transformed into foam cells via scavenger receptor phagocytosis of ox-LDL, forming the earliest lipid streaks of atherosclerotic lesions (82–88).

Ox-LDL induces an excessive production of reactive oxygen species (ROS) and reactive nitrogen species (RNS) *in vivo*, which induces antioxidant defense; however, the degree of oxidation exceeds the scavenging ability of oxides, resulting in tissue damage (89–92). SdLDL-C particles have the characteristics of a small size, low density, large surface area, long retention time *in vivo*, and induces great damage to endothelial cells, which leads to their increased permeability, chemotaxis of monocytes in blood vessels to form macrophages, and phagocytosis of oxidized LDL-C to form foam cells, thus further supporting the formation of AS (93). In addition, there are a few polar molecules on the surface of sdLDL-C particles, and their affinity with proteoglycans on arterial intima is enhanced, so they can easily adhere to the vascular wall and enter vascular endothelial cells, resulting in vascular endothelial damage and promoting the occurrence and development of AS (38, 94). This was verified by an experiment on the modification of LDL by methylglyoxal (MG). The modification of LDL by MG resulted in a significant decrease in the particle size of LDL similar to that of sdLDL. Vortex-stimulation showed that sdLDL-C showed higher PG aggregation rate and degree than unmodified LDL (95).

SdLDL-C not only injures the vascular endothelium but also activates the fibrinolytic system and produces plasminogen activator inhibitor 1 (PAI-1) (96–99) and vasoconstrictor thromboxane A2 (TXA2) (100, 101), thus promoting AS (93). A previous study found that plasma PAI-1 levels were positively correlated with the concentration of sdLDL-C (102). PAI-1 inhibits the function of t-PA and u-PA by binding to tissue-type plasminogen activator (t-PA) and urokinase-type plasminogen activator (u-PA) in a ratio of 1:1, The increase of PAI-1 expression *in vivo* will inhibit the normal fibrinolytic system, which is easy to lead to thrombosis (103). TXA2 activates TP receptors, activates RhoA/Rho kinase pathway through its G-protein coupled receptors, and increases calcium levels in hepatic stellate cells (HSC), resulting in vasoconstriction, platelet aggregation, thrombosis and atherosclerosis (104). Therefore, compared with LDL-C, sdLDL-C has a stronger effect on AS, and the damage it induces to the blood vessel wall lasts longer (105) (Figure 2).

## Anti-AS Interventions Involving SdLDL-C

### Non-medicinal Interventions

SdLDL-C levels are closely associated with meal compositions and dietary habits. Almonds have been shown to reduce LDL-C levels; however, there is limited data regarding their effects on dyslipidemia characterized by accrued levels of VLDL and sdLDL-C particles that are related to abdominal fat and high carbohydrate intake (106). A current meta-analysis of randomized controlled clinical trials found that consumption of almonds can reduce plasma TC concentration by 0.15 mmol/L, TG concentration by 0.07 mmol/L, and LDL-C concentration by 0.12 mmol/L (107). Studies have shown that almonds and almonds with dark chocolate and cocoa ingested for four weeks have a good effect on the levels of lipids, lipoproteins, and apolipoproteins, and the combined consumption of dark chocolate, cocoa, and almonds significantly reduces levels of sdLDL-C, apoB, and the ratio of apoB/apoAI, which in turn are expected to reduce the risk of CHD (108). Avocados are a nutritious source of monounsaturated fatty acids (MUFAs), which are rich in antioxidants. Avocados have an extra effect of reducing LDL-C levels, especially sdLDL-C particles, which are prone to oxidation in the body and are associated with an increased risk of CVD (109). In another randomized, crossover, controlled feeding study of patients with elevated LDL-C levels, compared with a daily intake of pistachios (32–63 g), a twice-daily intake of pistachios (63–126 g) significantly decreased sdLDL-C levels within four weeks. This experiment adopted a double-blind crossover design in which 30 postmenopausal women with moderate hypercholesterolemia were randomly assigned to two 35-day diets supplemented with corn oil or partially hydrogenated soybean oil to diets providing energy intake for weight maintenance. The results illustrated that the decrease in sdLDL-C concentration was positively correlated with a decrease in TG (110). In a randomized, double-blind, crossover study, subjects ate 10 grams of flaxseed oil or corn oil at dinner once a day, containing 5.49 and 0.09 g α-linolenic acid, respectively. Blood samples were collected at 0.4 and 12 weeks for the analysis of serum lipids, lipid-related proteins, serum fatty acids and serum sdLDL-C. Flaxseed oil supplementation significantly decreased the concentration of sdLDL at 4 and 12 weeks (111).

A study by Mendoza et al. strengthened the relationship between weight loss and reduced sdLDL-C levels. After weight loss, the concentration of apoC-III decreased, and the average BMI decreased from 27 to 25 kg m^2^, which was related to an increase in the peak particle diameter of LDL and a decrease in serum concentration of sdLDL-type (112). In addition to dietary intervention, exercise can effectively reduce the risk of cardiovascular disease (113). One hundred participants from the RESOLE trial (ages 50–70) were followed up for a year, starting with a three-week accommodation program that combined high exercise (15–20 h per week), diet restriction (500 kcal/day), and education. Forty age-matched healthy controls were recruited as a baseline reference. Lipoprint® electrophoresis was used to evaluate the distribution of lipoprotein subfractions in these subjects, allowing separation, and the results showed that sdLDL-C concentration decreased significantly after a 3-week residence plan (114). Another study conducted a six-month intervention on 30 hyperlipidemic subjects (12 males, 18 females; mean age, 64 years), focusing on moderate increases in physical activity. Clinical data before and after the intervention were observed. In addition to determining the average particle size of LDL and diacron reactive oxygen metabolites (d-ROMs) via gel electrophoresis, the risk factors for AS were also determined. The average LDL particle size after intervention was significantly larger than that before intervention (26.9 ± 0.3 vs. 27.1 ± 0.4 nm, mean ± SD, *P* < 0.01), whereas the level of sdLDL-C decreased significantly (115) (Table 2).

**Table 2:** Studies of non-medicinal intervention of sdLDL-C against AS.

**Intervention**	**Method**	**Subjects**	**Targets**	**Effect**	**References**
Diet	Almond	Atherogenic dyslipidemia	sdLDL-C, TC↓	Help in the maintenance of healthy blood lipid levels	(101)
	Almonds or dark chocolate	Overweight and obese individuals	sdLDL-C, LDL-C, TC↓	Improves lipid profiles	(103)
	Avocados	Overweight and obese individuals	oxLDL, sdLDL-C↓ plasma lutein↑	Reduce ox-LDL concentration and prevent AS	(104)
	Pistachios	Healthy adults	sdLDL-C, TG↓, HDL↑	Reduce cardiovascular risk	(105)
	Flaxseed oil	Healthy men	TC, LDL-C, ApoB SdLDL-C↓	Reduce sdLDL-C concentrations	(106)
Exercise	Weight loss or high carbohydrate	Overweight men	ApoB, ApoC, TG, sdLDL-C↓	Reduce sdLDL-C generation	(107)
	Physical exercise	Participants (50–70 years)	LDL-C, sdLDL-C↓, HDL↑	Improve carotid-intima-media thickness	(109)
	Moderate physical activity	30 hyperlipidemic subjects	d-ROM, sdLDL-C↓	Improve blood lipids	(110)

*SdLDL-C, small, dense low-density lipoprotein cholesterol; LDL-C, low-density lipoprotein cholesterol; HDL, high-density lipoprotein; oxLDL, oxidized low-density lipoprotein; TG, triglyceride; TC, total cholesterol; ApoB, apolipoprotein B; ApoC, apolipoprotein C; d-ROM, diacron reactive oxygen metabolites*.

### Medicinal Interventions

#### Statins

It is well-known that statins can effectively regulate blood lipid levels and delay the process of atherosclerosis. A study recruited 12 white men with metabolic syndrome. All subjects were treated with pitavastatin (4 mg/day) and their blood lipid levels were measured after 180 days. The results found that pitavastatin not only lowered LDL-C (−38%), sdLDL-C (sd-LDL4) and (sd-LDL5) are also effectively reduced (−27 and−33%, respectively). However, the sample size of this experiment is too small, and the sample size needs to be expanded to prove this point of view (116). A prospective, randomized, open-label, multicenter, parallel grouping comparative trial was conducted in Japan from October 2011 to November 2012. Eligible subjects (people at high cardiovascular risk over the age of 20) were treated with high-dose statins and conventional statins, respectively. The high-dose treatment group took 5 mg of rosuvastatin a day for the first four weeks, and then 10 mg a day for 8 consecutive weeks, and the low-dose statin group took 2.5 mg of rosuvastatin a day for 12 consecutive weeks. Lipid measurements were taken before, 4 and 12 weeks. The results show that both groups can reduce oxidized low-density lipoprotein cholesterol and sdLDL-C, and the effect of the high-dose group is more obvious (117). Although the high-dose group of statin therapy is effective in improving blood lipid levels, some cardiovascular events continue to occur. Therefore, high-dose statin therapy is recommended for the initial treatment of patients with high risk of atherosclerotic vascular disease (118). Another *in vitro* experiment evaluated the effect of combination therapy of Eicosapentaenoic Acid (EPA) and atorvastatin on endothelial cell function under oxidative stress conditions by measuring the release of NO and peroxynitrite (ONOO–) from human umbilical vein endothelial cells (HUVECS) to examine the comparative and time-dependent effects of these agents on endothelial dysfunction. Data shows that the combined treatment of EPA and atorvastatin can effectively reduce the level of sdLDL-C, thereby improving endothelial dysfunction, which may be because EPA contains substances that inhibit the oxidation of ApoB particles, which has a stronger antioxidant effect (119). However, some studies have shown that compared with patients who received atorvastatin for <6 days, patients who received atorvastatin for more than 90 days had significantly lower total cholesterol and LDL-C levels, but slightly lower sdLDL-C levels increased, but not significant (*p* = 0.06) (120).

#### Fibrates

Fibrates are agonists of peroxisome proliferator-activated receptor-α (PPAR-α), which regulate lipoprotein metabolism through transcription factors. Fibrates have shown effects in reducing fasting and postprandial TG and TG-rich lipoprotein residual particles (121). A meta-analysis of 13 studies illustrated that fibrates can reduce triglyceride levels, increase HDL-C levels, reduce the proportion of sdLDL-C, fibrates could be effective in secondary prevention considering a compound objective of non-fatal stroke, non-fatal myocardial infarction, and death of cardiovascular origin, and have fewer side effects; the most widely used drug of this class is fenofibrate (122). Other studies have also proved this point (123, 124). A retrospective study included 72 patients with type 2 diabetes. All patients received pemafibrate 0.2 mg (0.1 mg twice daily) for 24 weeks. During the entire study period, all patients did not change their exercise or diet regimens. The results show that Pemafibrate significantly reduces the levels of TG and sd-LDL-C, improves the composition of LDL and may reduce the risk of cardiovascular disease (125). It also has a better benefit-risk balance than conventional fibrates and can be applied to patients who find it difficult to use existing fibrates, such as those taking statins or those who have renal insufficiency (126).

#### Ezetimibe

Ezetimibe is a novel drug used for the treatment of dyslipidemia, which resists cholesterol absorption by inhibiting Niemann pick C1 like protein (NPC1L1) (127). Ezetimibe alone or in combination with statins can reduce the level of sdLDL-C (128). From October 2014 to November 2015, the author recruited patients with type 2 diabetes who had normal LDL-C levels and received statin therapy at the outpatient clinic of their institution. A total of 50 patients (31 men and 19 women) were enrolled in this study, and all subjects were randomly assigned to receive statins (statin group) or fenofibrate (160 mg/day) and ezetimibe treatment (10 mg/day), the results showed that the combination of fenofibrate and ezetimibe can effectively control the levels of sdLDL-C and TG, increase the level of HDL-C, and improve the vascular function of patients with type 2 diabetes. The effect of this combination is even better than treatment with statins alone (129). Therefore, to reduce the level of blood lipids using ezetimibe, it may be more beneficial if it is combined with other drugs. Current studies have illustrated that fenofibrate combined with ezetimibe can improve sdLDL-C levels and vascular function compared with statins (129).

#### Niacin

Niacin inhibits AS by activating the anti-inflammatory G protein-coupled receptor Gpr109a, also known as hydroxycarboxylic acid receptor 2 (HCA2), expressed on immune cells, inactivating the immune response and adventitious inflammatory cell infiltration (130). Niacin treatment was shown to decrease total cholesterol, triglyceride (20–50% decrease), and LDL-C levels. Additionally, niacin decreased sdLDL-C levels, leading to a shift to massive buoyant LDL particles, delaying the progression of AS (7). However, the negative effects of nicotinic acid were in accordance with the results of the HPS2THRIVE and AIM-HIGH trials, which suggests that its clinical application requires further study (131, 132).

#### Omega-3 Fatty Acids

Omega-3 fatty acids are essential fatty acids found in certain fish and vegetables. These are necessary for growth and development. Numerous studies have reported that omega-3 fatty acids scale back plasma triglycerides and increase HDL levels. They have been reported to inhibit blood platelet aggregation, improve endothelial function, decrease oxidative stress, and act as a potent medication agent (133). Changes in blood lipid and lipoprotein profiles were also observed after omega-3 fatty acid treatment for 8 weeks. The results of one study also showed that sdLDL-C levels decreased significantly after intake of omega-3 fatty acids (134). The omega-3 fatty acid EPA has substiantial antioxidant activity and can protect the membrane structure, which may promote scavenging of free radicals in sdLDL-C and membrane bilayers (135).

#### Other Western Medicine

Proprotein convertase subtilisin/kexin type 9 (PCSK9) belongs to the proprotein convertase family of enzymes that degrades LDL-R, which directly mediates the degradation of LDL-R in lysosome, which in turn increases plasma LDL level (136, 137). PCSK9 is positively correlated with sdLDL-C levels (138). At present, PCSK9 inhibitors (PCSK-9i) have been clinically used to reduce cholesterol levels and cardiovascular events in patients (139). In addition, resin and orlistat can also reduce sdLDL-C levels (140). Baricitinib treatment can also increase LDL levels and reduce sdLDL-C particles. One of the mechanisms by which baricitinib and related interventions increase the particle size of LDL-C may be the increased activities of phospholipase A2, liver lipase, lipoprotein lipase, and endothelial lipase. It has been reported that these enzymes are increased in a state of chronic inflammation (141).

## Conclusion and Perspectives

Lipid metabolism disorder is an important factor leading to AS. A large amount of evidence shows the pathogenic role of increased LDL-C in AS, and SdLDL-C, as a subgroup of LDL-C, has been proved to be a specific index for the detection of AS. Compared with traditional lipid monitoring, sdLDL-C monitoring has better sensitivity and specificity, and has better clinical value in predicting AS (7, 9). At present, there are many methods for the detection of sdLDL-C, but most of them have some limitations which cannot be widely used in the laboratory and clinic because they require expensive equipment, are time consuming, labor-intensive, and other reasons. The Lipoprint LDL system is currently the mainstream method for detection of sdLDL-C because it utilizes linear polyacrylamide gel electrophoresis to separate low-density lipoprotein according to particle size and charge, which has advantages of high efficiency, high speed, and low materials consumption (6).

Because of its small size and higher density compared to larger LDL-C particles, sdLDL-C has a greater ability to penetrate the artery wall, in addition to having a longer half-life and greater susceptibility to oxidative modification. Cardiovascular diseases caused by abnormal sdLDL-C are reflected in many clinical cases. SdLDL-C plays a variety of roles in the process of AS, such as affecting lipid metabolism, promoting the release of inflammatory factors leading to inflammatory reaction, releasing excessive ROS and RNS to produce oxidative stress, activating fibrinolytic system to produce thrombus. At present, there are non-drug interventions, such as regulating diet (low carbohydrates, soybeans, corn oil, etc.). Proper exercise can effectively improve the level of sdLDL-C in patients. Medical interventions, such as statins, fibrates, ezetimibe, niacin and omega-3 fatty acids, can reduce sdLDL-C levels in the body. The main mechanism is to improve the level of blood lipids and vascular endothelial function. In recent years, Pemabet and PCSK9 inhibitors have become the focus of current research as new interventions to prevent AS.

The specific mechanism of atherosclerosis caused by sdLDL-C has not been fully explained, and it is still being explored. Further exploration of the specific mechanism and intervention measures of sdLDL-C may provide a new direction for clinical prevention, evaluation and treatment of AS. Some studies have shown that the concentration of sdLDL-C is related to the changes of gene loci, and we speculate that gene detection may provide a new reference for the study of sdLDL-C. We know that intestinal microflora is significantly associated with lipid metabolism. A randomized controlled trial shows that changing intestinal microflora in patients with hyperlipidemia can effectively reduce sdLDL-C levels in patients with hyperlipidemia (142). However, the related research on the effect of intestinal flora on sdLDL-C is insufficient, whether it can reduce the level of sdLDL-C by improving intestinal flora, so as to reduce AS, is worthy of further study. In conclusion, these studies on the role of sdLDL-C in AS may provide information regarding new targets for the prevention and treatment of AS.

## Author Contributions

XJ and MW designed the article and wrote the manuscript. SY and JL searched the literature and aided in the design of the illustrations. All authors contributed to the article and approved the submitted version.

## Funding

The work was supported by the National Natural Science Foundation of China (Grant Nos. 81202805 and 82074254), the Beijing Natural Science Foundation (No. 7172185).

## Conflict of Interest

The authors declare that the research was conducted in the absence of any commercial or financial relationships that could be construed as a potential conflict of interest.

## Publisher's Note

All claims expressed in this article are solely those of the authors and do not necessarily represent those of their affiliated organizations, or those of the publisher, the editors and the reviewers. Any product that may be evaluated in this article, or claim that may be made by its manufacturer, is not guaranteed or endorsed by the publisher.
